# Mutual Authentication Scheme in Secure Internet of Things Technology for Comfortable Lifestyle

**DOI:** 10.3390/s16010020

**Published:** 2015-12-24

**Authors:** Namje Park, Namhi Kang

**Affiliations:** 1 Department of Computer Education, Teachers College, Jeju National University, 61 Iljudong-ro, Jeju-si, Jeju Special Self-Governing Province 690-781, Korea; namjepark@jejunu.ac.kr; 2 Digital Media Department, Duksung Women’s University, Ssangmoon-Dong 419, Dobong-Gu, Seoul 132-714, Korea

**Keywords:** IoT, Internet of Things, comfortable lifestyle, mutual authentication, IoT middleware

## Abstract

The Internet of Things (IoT), which can be regarded as an enhanced version of machine-to-machine communication technology, was proposed to realize intelligent thing-to-thing communications by utilizing the Internet connectivity. In the IoT, “things” are generally heterogeneous and resource constrained. In addition, such things are connected to each other over low-power and lossy networks. In this paper, we propose an inter-device authentication and session-key distribution system for devices with only encryption modules. In the proposed system, unlike existing sensor-network environments where the key distribution center distributes the key, each sensor node is involved with the generation of session keys. In addition, in the proposed scheme, the performance is improved so that the authenticated device can calculate the session key in advance. The proposed mutual authentication and session-key distribution system can withstand replay attacks, man-in-the-middle attacks, and wiretapped secret-key attacks.

## 1. Introduction

The Internet of Things (IoT) is a concept that describes a totally interconnected world in which devices of every shape and size are manufactured with “smart” capabilities that enable them to communicate and interact with other devices, exchange data, make autonomous decisions, and perform useful tasks based on preset conditions. It also refers to a world where technology will make life richer, easier, safer, and more comfortable. The IoT has begun to shape our modern world, where smart devices interact not only with humans, but also with other smart devices, objects, environments, and infrastructure [1]. Connected Intelligence is the technology that enables the IoT, bringing it to life and delivering intelligence everywhere, enabling designs and whole systems to be scaled and interact seamlessly. We believe that the greatest opportunities within the IoT will be in the transformational shift arising from the computing nexus to highly intelligent nodes, where intelligence is significantly scaled, and where nodes have the power to learn, adapt, and communicate.

Machine-to-Machine (M2M) communication provides each component (machine) with access to the Internet, leading to the evolution of the IoT technology. The IoT, which can be regarded as an enhanced version of M2M communication technology, was proposed to realize intelligent thing-to-thing communications by utilizing Internet connectivity. In the IoT, “things” are generally heterogeneous and resource constrained. In addition, such things are connected with each other over low-power and lossy networks (LLNs) [2,3,4,5].

In the IoT, several devices provide the communication service using the Internet protocol. In this environment, security is a critical element that is necessary to enable various types of applications and services. Various types of authentication technologies and session-key distribution/agreement technologies have been proposed for security services under M2M or sensor-network environments. As M2M evolves into IoT, the IETF Constrained RESTful Environments (IETF CoRE) working group is reviewing the lightweight datagram transport layer security (DTLS) for the IoT environment. The following sections provide examples of authentication technologies that have been proposed and the technology proposed by the standardization group [6,7,8,9].

In this paper, we consider an inter-device authentication and session-key distribution system for devices with only encryption modules. In the proposed scheme, unlike existing sensor-network environments where the key distribution center distributes the key, each sensor node agrees with the generation of session keys. In the proposed scheme, the performance is improved so that the authenticated device can calculate the session key in advance. The proposed mutual authentication and session-key distribution system is robust to replay attacks, man-in-the-middle attacks and wiretapped secret-key attacks. In this regard, an IETF standard group proposes to use a lightweight version of the DTLS protocol to support security services in IoT environments. However, the protocol cannot cover all of very constrained devices. To solve this problem, we propose a scheme that supports mutual authentication and session-key agreement between devices, and which contains only a single crypto-primitive module such as a hash function or cipher function because of its resource-constrained property. The proposed scheme enhances the performance by pre-computing a session key, and is able to withstand a variety of attacks.

## 2. Review of Secure IoT Environment

The IoT service middleware platform should satisfy the following requirements in order to provide the function of acquiring and analyzing sensor-network monitoring information or sensor data periodically and continuously in the links of multiple heterogeneous sensor networks for IoT application services.

### 2.1. User Authentication and Authorization

The IoT service middleware platform should provide authentication for the multiple IOT application services registered in the platform when they request access to the platform, and authorization to allow the requested service functions to access certain sensor networks. The detailed requirements are as follows:

(1) It should be possible to register the IoT application service as a user that is uniquely identified in the platform.
(2) Each user should be able to select either the ID-based method or authentication-certification-based method, and the platform should be able to perform authentication using the method selected by the user.
(3) In the case of authentication failure, proper error messages should be issued.
(4) It should be possible for a user in the platform to grant access to the registered platform service.
(5) When using the platform service for which authentication is declined, proper error messages should be issued.
(6) The user in the platform should be able to authorize user to access the registered sensor network.
(7) The platform should be able to perform appropriate authentication to ensure legal use in the case where the user uses the sensor network via the service function.
(8) When using the sensor network for which authentication is declined, proper error messages should be issued.
(9) The platform should be able to handle the release request of the IOT application service for access to the platform.

### 2.2. Platform Session Management

The IoT service middleware platform should provide the session management function. This enables it to generate and remove the application sessions for the application services after obtaining authorization from the platform, and it functions to generate and remove service sessions for the service functions of the platform. The detailed requirements are as follows [10,11,12,13]:

(1) The platform should be able to generate new application sessions for authorized application services.
(2) The platform should be able to provide a unique identifier to the application session of an authorized application service.
(3) The platform should be able to maintain the application session continuously and to remove it given an explicit request including the application session identifier.
(4) The platform should be able to generate a service session using the platform service for an effective application session.
(5) The platform should be able to provide a unique session identifier to the generated service session.
(6) The platform should be able to remove the service session using the session identifier for the service session.
(7) The platform should remove all service sessions related to the application session when the application session is removed.

### 2.3. Service Message/Notification

The IoT service middleware platform provides the processed results by responding to the requests for processing the service function. However, it cannot instantly respond to such requests by the application service because the sensor-network state and sensor data from the sensor network are collected and processed continuously or periodically. Therefore, the processed results should be provided asynchronously against the requests of the application service, and should be classified as two types: message and notification. The message is generated when the platform performs and generates the data-processing results for the data collected from the sensor network. The notification is generated when abnormal states or events are detected by imposing certain conditions on the sensor network, the platform, and the results of the service functions. The detailed requirements are as follows:

(1) The platform should be able to provide the processed results by instantly responding to the service function request of the authorized application service.
(2) The platform should be able to provide asynchronous messages continuously or periodically in the case where the results of the service function request of the authorized application service are generated continuously or periodically.
(3) The platform should be able to provide notifications periodically or continuously when certain types of changes and detections corresponding to specific conditions occur in the sensor networks, platform, and service functions that are requested by the authorized application.
(4) The application service should register the message destination to enable it to receive messages or notifications from the platform, and such destination information should contain the receiving method (JMS, JDBC and RMI), receiving address (URL or IP address and port information), and receiving information (parameters according to the receiving methods), *etc*.
(5) The application service can request a notification by designating the notification type that is registered in the platform. Of the recognized notification types, the platform should be able to provide only the notification that is requested by the application service.
(6) The platform should be able to generate the unique identifier of the message or notification according to the receiving destination registration of the application service, and should provide it to the application service.
(7) The platform should be able to send a notification message regarding the registration of a message or the notification destination of the application service.
(8) The platform should be able to explicitly cancel the registration of a destination using the directory or notification-destination identifier based on the requests by application services.
(9) The platform should be able to delete the registration of a message or notification destination according to the deletion of application sessions or service sessions.

### 2.4. Sensor Network Monitoring and Control

The IoT service middleware platform should be able to monitor the changes to the state information of the multiple sensor networks that are linked to the platform, and to control the operation of the sensor networks by detecting the requests of the application services or abnormalities in the sensor networks. The detailed requirements are as follows [14,15,16]:

(1) The platform should be able to acquire the state information of sensor networks continuously by performing the sensor network monitoring operation after the sensor networks are normally linked to the platform.
(2) The platform should be able to update the information registered in the IoT metadata when the state information of sensor networks is changed, and to send notifications of changes in the state of a sensor network to the application service that requests sensor-network notification.
(3) The application service should be able to control the authorized sensor networks using commands such as start, suspend, and stop the monitoring operation.
(4) The application service should be able to change the monitoring cycle for the authorized sensor network.

### 2.5. Sensor Data Acquisition/Processing

The IoT service middleware platform should be able to acquire sensor data continuously from the multiple sensor networks linked to the platform. This is in order to provide the application service with the data, and to provide the application service with the processed results of the acquired sensor data that were requested by the application service. The detailed requirements are as follows:

(1) The platform should be able to simultaneously handle the sensor data query requests by multiple application services in order to acquire sensor data from the multiple registered sensor networks, including the sensor or actuator.
(2) The platform should be able to handle the event query requests by providing the results to the application services when specific conditions are satisfied.
(3) The platform should be able to handle the continuous query requests by providing the sensor data that are acquired and processed according to the given time period and cycle.
(4) The platform should be able to handle the stream query requests by providing the sensor data that are acquired and processed continuously according to the time window and moving slice.
(5) The query process requests of application services should be possible by the request interface including command lines that have grammar that is similar to that of SQL, or by the request interface based on parameters by stag.
(6) In the case of query process requests made by application services, the sensor-network identifier should be designated as the unique identifier following the IoT metadata standard or in the alias name, and the types of sensors should be designated in the sensor-type name.
(7) In the case of query process requests made by application services, each query should include the processing of aggregate functions such as max, min, avg, and count.
(8) In the case of query process requests made by application services, a conditional equation should be included to limit the sensor values.
(9) In the case of query process requests made by application services, the processing of logical sensor networks should be supported to logically bind more than two sensor networks to be a single identifier.
(10) The platform should be able to provide a query identifier that is uniquely recognized in the query requests.
(11) The platform should be able to control the query processes that are performed continuously or periodically by the application services. This may be achieved by suspending, resuming, and stopping them.

### 2.6. Authentication and Session-Key Distribution between IoT Devices

The sensor-network environment and IoT are similar to each other in that they are both lightweight sensor-network environments. In existing sensor-network environments, communication is between sensor nodes and the infrastructure node (base station), which is different from the communication between sensors in an IoT environment. In particular, the devices comprising the web-of-things (WoT), which falls under the category of IoT, can simultaneously conduct both web-client functions and web-server functions. Because of this difference, it is difficult to apply the typical sensor-network-based method as is to the IoT environment [17,18,19,20].

Some of the existing studies have proposed the method of shared-key setting and session-key distribution through the base station and the key distribution center, respectively. However, this technology requires communication between a sensor and the base station to share the secret key, followed by communication between the base station and the key distribution center. The use of both encryption and hash functions can be a burden for resource-limited sensors, where not all of the modules can be mounted.

The key distribution method using public keys in sensor-network environments has been proposed. Again, for the above reason, it is difficult to apply this method to a light sensor where encryption modules such as RSA and elliptic curve cryptography (ECC) cannot be mounted.

### 2.7. Lightweight Security Technology for IoT Environments

The IETF CORE working group for the standardization of IoT technology is currently considering the application of TLS, DTLS, IPSec, HIP, and PANA, which have been adopted in TCP/IP-based Internet environments. In particular, the basic concept is to apply DTLS to CoAP, which is the key protocol. In order to apply existing IP-based security protocols, a plan is required for lightweight devices considering the computational capabilities and the available memory space. Further, fewer messages should be transmitted considering the communication capability of LLN. For this purpose, various technologies are proposed. The DTLS protocol can be made lightweight by reducing the number of packets comprising the handshake message or by simplifying the verification of authentication certificates.

## 3. Proposed Security Scheme

In this paper, we propose the categorization of resource-limited sensors in the WoT environment proposed by the Lightweight Implementation Guidance (LWIG) working group as Class 0 super-light sensors and Class 1 or above sensors. Further, we propose the appropriate application of the authentication method and the session-key sharing method for the performance of each group of sensors. The parameters used in the proposed system are as follows:

- I is the authentication initiator.
- R is the responder to the message sent by the authentication initiator.
- r1 and r2 are random nonce values.
- k*IR* is the key that is securely shared in advance between the authenticator and the responder.
- sk is the session key that is shared between the authenticator and the responder.

### 3.1. Symmetric-Key-Based Authentication and Session-Key Agreement Scheme

The proposed system enables inter-device authentication and the session-key sharing method for resource-limited devices that can use only symmetric-key-based encryption modules. This method assumes that k*IR* is the secret key value, and E_k(x), which is the encryption function, has been securely saved at the device-setting stage. The procedure for authentication and session-key agreement is as follows:

**[Inter-device authentication and session-key sharing procedure]**

(1) I → R: {I_ID, r1}
  I generates a random number r1, and sends it with I_ID, which is its own ID, to R.
(2) R → I: {R_ID, E_k*IR* (r1 || r2)}
  R encrypts the received r1 and the coherently generated r2 with k*IR*, and sends the value with R_ID, which is its own ID to I. (3) I, R: sk = E_k*IR* (r1 ⊕ r2).
(3) I and R XOR-operate r1 and r2, and encrypt the value with k*IR* to generate the session key sk.
(4) I → R: {E_sk(r2)}
  I encrypts r2 with the session key sk, and retransmits it to R to get authentication.

**[Data encrypted communication]**

(5) R → I: {E_sk(Data1)}
  R authenticates I with the message (4), and encrypts the data with sk before transmitting it.
(6) I → R: {E_sk(Data2)}
  I uses sk for the encryption of data.
(7) Session End
  The session ends when the encrypted communication is finished, and the two entities dispose the sk.

With this method, the authentication requestor I and the responder R can authenticate each other, and they can share a different session key for each authentication session without the need to exchange new parameters or adopt an additional method because the random value used for authentication is also used for the session-key agreement. After transmitting the message (2), the responder R calculates the session key using r1, r2, and E_k*IR* to immediately verify the message (3), increasing the calculation efficiency. R encrypts r1 and coherent r2 to transmit the message (2). Therefore, different methods are used for the XOR operations of r1 and r2 that are used to generate the session keys and the input value. Because the values of (2) and (3) are different, even if the same r1 and r2 are used, this method can protect the data against message-modulating attacks by third parties [21,22,23,24,25].

### 3.2. Hash-Based Authentication and Session-Key Agreement Scheme

This section describes the case in which a Class 0 device has a hash function only because of limited resources. The proposed system provides the methods for inter-device authentication, the sharing of session keys, and the authentication of data transmission. This method assumes that k*IR*, which is the secret key value, and h(x), which is the hash function, have been saved securely in the device-setting stage. The encryption strength is based on AES 128 bit (*i.e.*, the hash output is assumed to be 256 bits, and the length of the random number is assumed to be 128 bits). The procedure for the mutual authentication and authentication of the data transmission is as follows:

**[Mutual authentication and session-key procedure]**

※ KH (data) = h(k*IR*_1⊕r1||h(k*IR*_2⊕r2||data))

(1) I → R: {I_ID, r1}
  I sends its own ID and r1 to R, the target device for authentication.
(2) R → I: {KH(r1), r2}
  R divides the 256-bit key kIR shared in advance with I by 128 bits into kIR_1 (the first half) and kIR_2 (the second half). Dividing a kIR into kIR_1 and kIR_2 has the effect of generating 2 keys. R XOR operates on kIR_2 and r2, and the hash operates on the value and the coherent r1. R uses this result and the coherent result of the XOR operation of kIR_1 and r1 as the input values of the hash function. Then, R sends the result of the hash operation, the ID of R, and the coherently generated nonce value r2.
(3) I → R: {KH(r2)}
  I verifies whether the received message (2) is the response to the message (1), XOR-operates kIR_2 and r2, and the hash then operates on the XOR operation result with the coherent r2. I hash operates on the value and the coherent XOR value of KIR_1 and r1, and sends the result.
(4) I, R: sk = {|h((r1 || r2) ⊕ k*IR*)|128}
  I and R use the random nonce values r1 and the coherent r2 used for mutual authentication, XOR-operate and hash-operate with the shared key k*IR*, and use the 0th bit~127th bit as the session key sk.

**[Data transmission by transmitting authenticated message]**

※ SKH(data) = h(sk⊕r1||h(sk⊕r2||data))

(5) R → I: {Data1||SKH(Data1)}
  R XOR operates on the generated session key sk and r2, the hash operates on the coherent data to be transmitted, the hash operates on the result with the session key, and r1 with the XOR value, and transmits the transmission authentication value with the coherent data 1.
(6) I → R: {Data2||SKH(Data2)}
  I XOR operates on the generated session key sk and r2, the hash operates on the coherent data to be transmitted, the hash operates on the result with the session key, and r1 with the XOR value, and transmits the transmission authentication value with the coherent data 2.
(7) Session end
  The session ends when data transmission is finished.

The proposed system does not provide an encryption protocol, and uses r1 and r2 as the ipad and opad of the hash message authentication code (HMAC), respectively, providing mutual authentication between lightweight devices with no HMAC and the data-transmission authentication for the transmitted data. Using a 256-bit key, which is longer than the result bit of the provided hash function, the system provides the same security level as HMAC. As the system generates a session key with a random nonce value, the entities can share a new session key for each authentication session. The responder R can generate the session key in advance after transmitting the message (2), sending the transmission authentication message immediately for the data to be transmitted.

### 3.3. Comparative Analysis

#### 3.3.1. Authentication and Session Key Distribution between IoT Devices

The sensor network environment and IoT are similar to each other in that they are the light-weight sensor network environment. The communication under the existing sensor network environment is between sensor nodes and the infrastructure node (base station), which is different from the communication between sensors under the IoT environment. Especially, the devices composing WoT which falls under the category of IoT can conduct both the web client function and the web server function at the same time. Due to this difference, it is difficult to apply the typical sensor-network-based method as it is to the IoT environment [17,18,19,20].

Some of the existing researches have proposed the method of shared key setting and session key distribution through the base station and the key distribution center. However, the technology requires a communication between a sensor and the base station to share the secret key, and then, a communication between the base station and the key distribution center. Using both encryption and hash function can be a burden for resource-limited sensors where not all the modules can be mounted.

The key distribution method using the public key under the sensor network environment has been proposed. Again, due to the above reason, it is difficult to apply the method to a light sensor where the encryption module, such as RSA and ECC, cannot be mounted.

#### 3.3.2. Lightweight Security Technology for the IoT Environment

The IETF CORE working group for standardization of the IoT technology is considering application of TLS, DTLS, IPSec, HIP and PANA, which have been adopted in the TCP/IP-based Internet environment. Especially, the basic concept is to apply DTLS to CoAP, which is the key protocol. In order to apply the existing IP-based security protocol, a plan for lightweight device is required in consideration of the calculation capability and the memory space. Also, the messages transmitted must be minimized in consideration of the communication capability of LLN. For this purpose, various technologies are proposed. The DTLS protocol can be light-weighted by reducing the number of packets of the handshake message or by simplifying verification of authentication certificates.

This paper proposes to divide the resource-limited sensors under the WoT environment presented by the LWIG working group into the Class 0 super-light sensors and the Class 1 or above sensors, and to apply the authentication method and the session key sharing method appropriate for the performance of each group of sensors. The proposed system does not provide an encryption protocol, uses r1 and r2 as ipad and opad of HMAC, providing the mutual authentication between lightweight devices with no HMAC and the data transmission authentication for the data transmitted. Using a 256 bit key, which is longer than the result bit of the provided hash function, the system provides the same security level as HMAC. As the system generates a session key with a random nonce value, the entities can share new session key for each authentication session. The responder R can generate the session key in advance after transmitting the message (2), sending the transmission authentication message immediately for the data to be transmitted.

### 3.4. Security Analysis of Proposed Scheme

The proposed system can withstand replay attacks, man-in-the-middle attacks and wiretapped secret key attacks. Attackers can intercept packets that are transmitted during the authentication and distribution of the session key between sensor nodes, and then replay them after some time. Because the system proposed in this paper generates a new nonce value for every authentication attempt, no authentication is established if an attacker replays the packets after a time interval [26,27,28,29,30].

For the system described in Section 3.1, the entities use a new session key after a set time to prevent replay attacks. For the system described in Section 3.2, a new session key changes the message-transmission authentication value, preventing replay attacks.

A third party may participate in the authentication and session-key distribution between sensor nodes in order to pass through the authentication process, or to acquire hidden information to attempt man-in-the-middle attacks. In the proposed system, a third party may pass through the authentication by forwarding a simple message, but cannot generate a session key because the attacker has no secret key shared in advance.

For the system in Section 3.1, the attacker can neither decrypt nor generate encrypted data. For the system in Section 3.2, the system can respond to man-in-the-middle attacks because the attacker cannot falsify or generate the message-transmission authentication value.

Attackers may collect information that is exchanged for the generation of a session key in order to attempt to generate the session key. For the proposed system described in Section 3.1, because the random nonce value, r2, is encrypted before transmission, attackers must know both the master key that is shared in advance and the r2 value in order to generate a session key.

For the system in Section 3.2, the attackers cannot generate a session key because they the master key value is unknown. Therefore, the system proposed in this paper can provide data secrecy and transmission authentication by using session keys.

#### 3.4.1. Man in the Middle Attack

A Man in the Middle attack happens when an attacker secretly relays and possibly alters the communication between two parties who believe they are directly communicating with each other. This is one of the most used attack schemes in wireless networks. In the case of the proposal, a Man in the Middle attack cannot be carried out because there is no way to gain information during the transaction. In particular, the scheme uses a single beacon to send some information from the sender to the receiver. Therefore, there is no communication between them, so that other users cannot intercept communication and hence cannot impersonate communications. If the scheme is used to establish a secret key through the Diffie-Hellman algorithm, thanks to the fact that the protocol uses strong mutual authentication with secret keys, it is robust against a Man in the Middle attack. Mutual authentication refers to two parties authenticating each other at the same time.

#### 3.4.2. Other Attacks

In proposed protocol, due to the lack of a centralized structure, Denial of Service (DoS) attacks can be frequent. In order to protect the proposed scheme from DoS attacks, although the communication produced with the application is through a non-secure channel, only legitimate nodes of the networks are able to send and decrypt valid messages. Another dangerous attack in protocol is the sibling attack, which occurs when a node uses illegitimately multiple identities. This problem is avoided in the proposed scheme thanks to the distributed nature of the used other protocol. Finally, the proposal is also resistant to identity theft because node access is controlled by a strong protocol.

## 4. Service Flow of IoT Service Middleware Platform

### 4.1. Flow of Platform Operation

**Figure 1:**
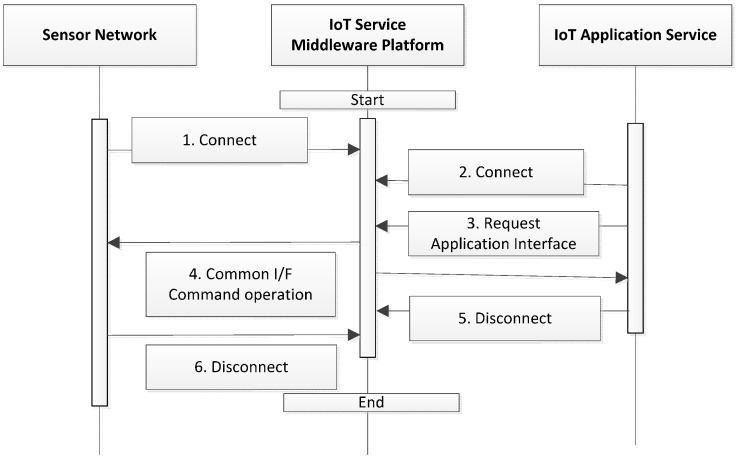
Operating flow of the platform.

The flow of platform operation is shown in Figure 1 above. The platform enters the operational state after the initial actuation, and a link is then established to the sensor networks and application services. Multiple sensor networks are linked by the connection requests in the operational state of the platform, and the application services are then also linked to the platform in its operational state. The platform linked to the sensor networks processes the commands continuously according to the common-interface standard based on service requests obtained by application services, and each application service performs its actions using the application interface with the platform. Connections between the applications and sensor networks are possible. Finally, the platform stops functioning as a platform in the finish stage (Figure 1).

### 4.2. Flow of User Authentication/Authorization

The authentication process that is performed during the request by an application service, and the flow of authentication by the service request are shown in the figure above. After the platform is activated and linked to the sensor networks, the application service should first request authentication in order to obtain the platform service. The authentication is realized when the application service sends the generated credential to the platform, and the platform -interprets the credential and checks whether or not the application service is a registered user. The credential should be treated confidentially, and can be generated selectively using an ID/Password-based authentication method and a certificate-based authentication method. The platform generates application sessions when the authentication is confirmed, and sends the Session ID, which is the result of the authentication, to the application service.

**Figure 2:**
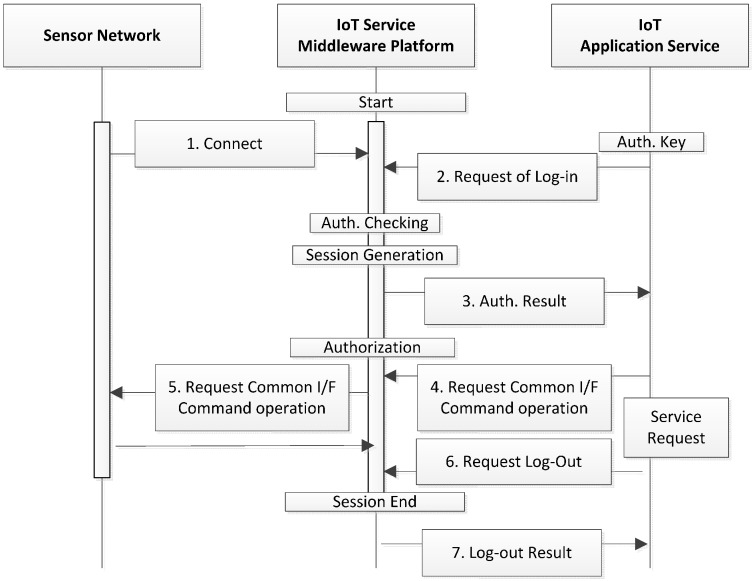
Authorization process of the application service.

After the authentication process, the application service sends requests using the service interface provided by the platform. These requests enable the platform to carry out the authorization process that determines whether the services or resources provided by the platform can be used, and the results obtained in the sensor networks are provided to the application services. The basic services provided by the platform include sensor network control, sensor network monitoring, and sensor data query processing, and may be expanded to include other types of services. The resources provided by the platform refer to the sensor networks that are registered on, and that are linked to the platform. The platform should maintain the information regarding permissions of services and resources allocated to each user for the purpose of the authorization process. The application service is disconnected by sending a log out request to the platform (Figure 2).

### 4.3. Flow of Service Session Management

The ubiquitous sensor network (USN) service middleware platform provides groups of functions to the applications services, and defines those groups as the platform service. In other words, application interfaces that are provided by abstracting the sensor-network functions, multiple application interfaces that are provided for monitoring sensor networks, and query process interfaces for the acquisition and analysis of sensor data should be used together with the related interface in each interface group. The processed results obtained according to each service request can be transmitted to the application services in various ways depending on the characteristics of each service.

The process flow of service sessions that are generated and removed in the platform for the application services are shown in the figure above. First, the sensor networks are connected to the platform. The platform generates application sessions after authentication of the application service, and the application service performs requests for the generation of service sessions (S2, S3) prior to the request for the platform service. The application service can generate multiple service sessions for the services provided by the platform. The application service makes requests (S4) via the service-application interface within the generated service session. Finally, the application service should request the removal of service sessions prior to the requests for the removal of application sessions, and the platform should remove the remaining sessions for the service upon receiving the requests for removal from the application service.

### 4.4. Flow of Message and Notification

The service results for the application service requests are defined as a message and notification. The message is not the returned value of the processed results that are instantly transmitted in response to the service requests. However, it is the result type that is generated for transmission to the application service after continuously processing the requests. In other words, it does not include the results transmitted synchronously in response to the application service requests, but is the result type generated by the process in the platform and which is transmitted asynchronously. The notification is the result type that is pushed to the service when events occur on the platform, or when changes in state are detected. The notification is different from the message in that the notification type and notification contents (reasons) are both included and transmitted.

**Figure 3:**
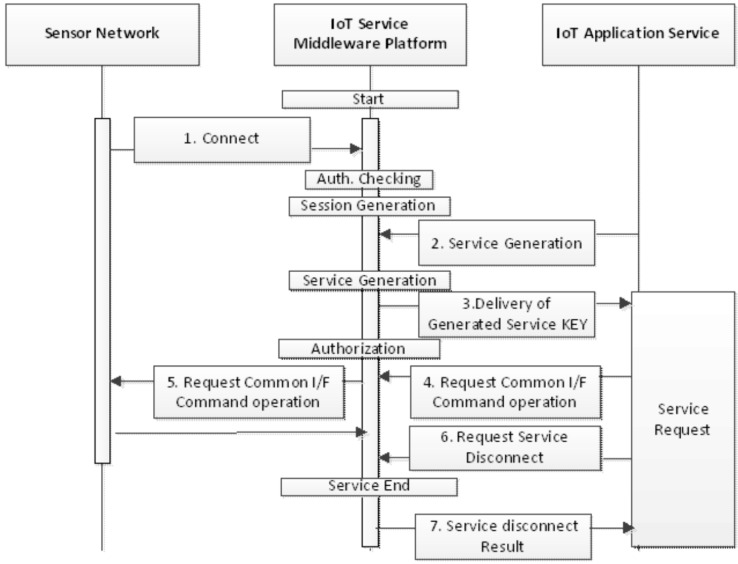
Flow of the platform service session management.

The transmission flow of the message and notification to the application service is shown in Figure 3. First, the sensor network is linked (S1) to the platform. The application can register the subscription URI by requesting the subscription of the message or notification to each service session. Once the subscription URI is registered, the platform transmits messages and notifications continuously whenever they are generated as the processed results for the service requested by the application. The application service can then change and remove the subscription URI. Once the application service removes the subscription URI, the platform does not send the processed results to the subscription URI, and discards them.

### 4.5. Flow of Sensor Network Monitoring and Control

**Figure 4:**
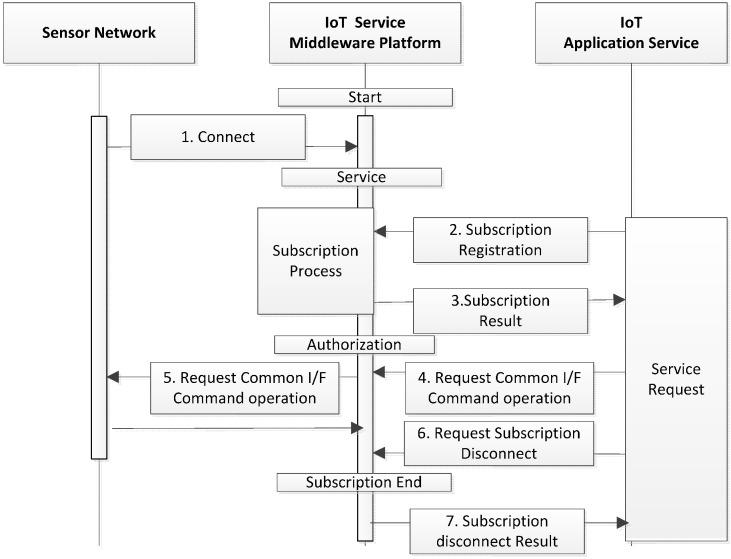
Flow of the message and alert process.

The flows of the sensor-network monitoring and control are shown in Figure 4. The sensor networks linked to the platform transmit the monitoring information periodically via the common interface of the sensor network. The platform dynamically updates the monitoring information of the multiple sensor networks that are linked to the platform, and maintains them for use in the related application or platform. The USN application service obtains the monitoring-state information from the platform in the form of a notification. When there are changes to the monitoring-state information, the platform sends a notification to the notification subscription URI of the registered service, if necessary. The application service then uses the application interface to transmit control requests such as changes in the sensor-network monitoring cycle, transmission method, and On/Off of the network elements, and the platform gives control commands to the sensor networks and transmits the results to the service.

### 4.6. Query Process of Sensor Data and Actuator

The sensor data query is a request that is sent by the application service in order to obtain the sensor data acquired from the sensor networks in the format preferred by each application [31,32,33,34]. The flow of the sensor data query process is shown in Figure 4. First, the sensor network is linked to the platform. Then, the USN application service requests the query process in the query statement that is expressed based on the query type and query syntax that is supported by the USN service middleware platform. The platform provides the identifier of the query process request to the service, and then transmits to the service the results of the query process for the sensor data that are acquired from the sensor network. The application service controls the query by sending control requests such as suspend, resume, and stop for consecutively performed queries such as the continuity query or stream query. The USN service middleware platform should support the following types of sensor data query.

## 5. Comparison with Related Work

**Figure 5:**
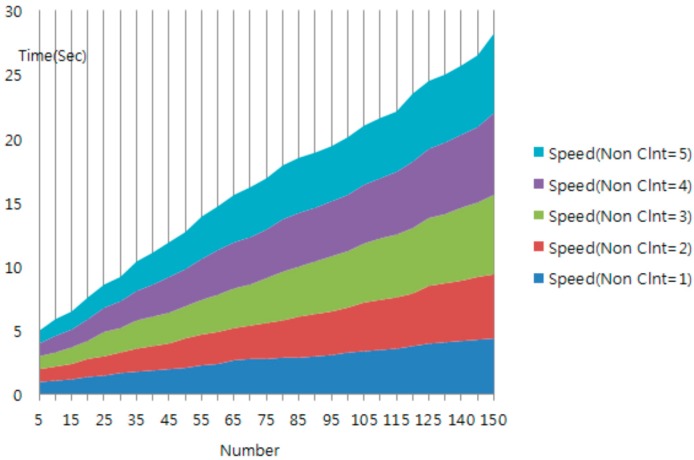
Simulation result for J-IoT middleware filtering.

Figure 5 showed difference for 4 s that compare average transfer time between client and mobile IoT middleware of middleware filtering and unfiltering by network. Generally, as the tag number increased, showed phenomenon that increase until 4 s [35,36,37,38,39,40].

**Figure 6:**
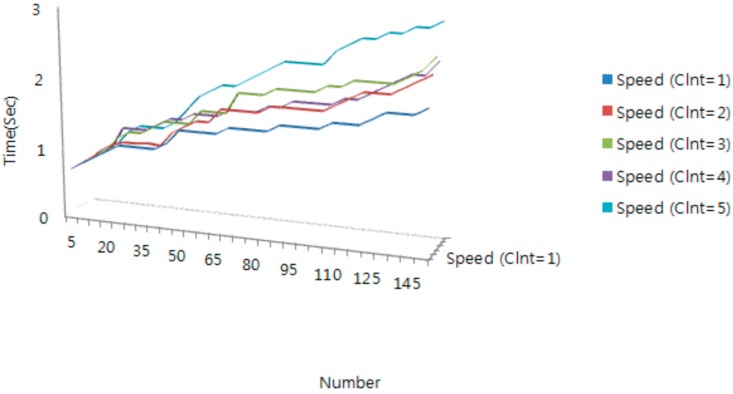
Simulation result for mobile IoT middleware non-filtering.

Figure 6 shows the average transmission time with increasing tag number in a non-filtering protocol environment. As the tag number increases, we observe that the average transfer time generally increases. Moreover, the average transfer time increases rapidly in the case where the tag number is greater than 45. Therefore, about 40 IoT devices can be simultaneously processed stably by a PC in a testbed environment. When we compare the filtering time and protocol time, the time of mobile IoT middleware platform’s filtering module is occupying and shows the importance of the signature module for about 32% of the entire protocol time.

## 6. Conclusions

The IoT, which can be regarded as an enhanced version of M2M communication technology, was proposed to enable intelligent thing-to-thing communications by utilizing Internet connectivity. In the IoT, “things” are generally heterogeneous and resource constrained. In addition, such things are connected with each other over LLNs. In this paper, we focused only on inter-device authentication and the session-key distribution system for devices with only encryption modules.

In the proposed scheme, unlike existing sensor-network environments where the key distribution center distributes the key, each sensor node is involved with the generation of session keys. In the scheme, the performance is improved so that the authenticated device can calculate the session key in advance. The proposed mutual authentication and session key distribution system can withstand replay attacks, man-in-the-middle attacks and wiretapped secret-key attacks. However, the proposed system assumes that the devices involved securely share k*IR*. If k*IR* is compromised, there is a limit to the security that can be guaranteed by the proposed system. Like many existing proposed systems using preset secret keys, in this paper, the proposed system assumes secure channels and storage methods. However, further research is required to realize the secure sharing of k*IR.*
